# Novel Broad Spectrum Inhibitors Targeting the Flavivirus Methyltransferase

**DOI:** 10.1371/journal.pone.0130062

**Published:** 2015-06-22

**Authors:** Matthew Brecher, Hui Chen, Binbin Liu, Nilesh K. Banavali, Susan A. Jones, Jing Zhang, Zhong Li, Laura D. Kramer, Hongmin Li

**Affiliations:** 1 Wadsworth Center, New York State Department of Health, 120 New Scotland Ave, Albany, NY, 12208 United States of America; 2 Department of Biomedical Sciences, School of Public Health, State University of New York, Albany, PO Box 509, New York, 12201, United States of America; University of Berne, SWITZERLAND

## Abstract

The flavivirus methyltransferase (MTase) is an essential enzyme that sequentially methylates the N7 and 2’-O positions of the viral RNA cap, using *S*-adenosyl-L-methionine (SAM) as a methyl donor. We report here that small molecule compounds, which putatively bind to the SAM-binding site of flavivirus MTase and inhibit its function, were identified by using virtual screening. *In vitro* methylation experiments demonstrated significant MTase inhibition by 13 of these compounds, with the most potent compound displaying sub-micromolar inhibitory activity. The most active compounds showed broad spectrum activity against the MTase proteins of multiple flaviviruses. Two of these compounds also exhibited low cytotoxicity and effectively inhibited viral replication in cell-based assays, providing further structural insight into flavivirus MTase inhibition.

## Introduction

The genus *Flavivirus* in the family *Flaviviridae* is composed of about 53 arthropod-borne viruses [1–3]. The four serotypes of dengue virus (DENV), yellow fever virus (YFV), West Nile virus (WNV), Japanese encephalitis virus (JEV), and Tick-borne encephalitis virus (TBEV) are categorized as global emerging pathogens that can cause serious human disease, including meningitis, myelitis, encephalitis, and hemorrhagic disease [4–7]. DENV infection threatens approximately 2.5 billion people around the world. Since 1999, WNV has spread rapidly throughout the Western Hemisphere, including the contiguous United States, Canada, Mexico, the Caribbean, and into parts of Central and South America [8]. Although vaccines for humans are currently available for YFV, JEV, and TBEV [6, 7], no clinically approved vaccine or antiviral therapy for humans is available for WNV and DENV. Therefore, it is a public health priority to develop and improve vaccines and antiviral agents for prevention and treatment of flavivirus infections.

The flavivirus genome is a positive (or sense) single stranded RNA with a type I cap at the 5’ end followed by the conserved dinucleotide sequence 5’-AG-3’ [2, 9, 10]. The viral genome encodes a polyprotein that is co- and post-translationally processed by viral and cellular proteases into three structural proteins (capsid [C], premembrane [prM] or membrane [M], and envelope [E]) and seven nonstructural proteins (NS1, NS2a, NS2b, NS3, NS4a, NS4b, and NS5) [11]. Several of these proteins are targeted for drug development [2, 12–20]. Particularly, the flavivirus NS5 methyltransferase (MTase) recently became an attractive target for therapeutic inventions [2, 14, 15, 21–30]. Flavivirus NS5 MTase performs both N7 and 2’-O methylation of viral RNA cap [10, 31, 32]. Recombinant MTases from various flaviviruses sequentially generate GpppA → m^7^GpppA → m^7^GpppAm, using S-adenosyl methionine (SAM) as the methyl donor. Upon completion of methylation reaction, SAM becomes S-adenosyl homocysteine (SAH), and gets released from the MTase. The N7 methylation of the viral mRNA cap is an essential step in the virus life-cycle, as defects in N7 methylation abolished DENV, WNV, YFV, and Kunjin virus replication [10, 33–38]. We and others reported that sinefungin (SIN) and several nucleoside analogues could inhibit the MTase activity and virus replication [21, 30, 34]. An additional flavivirus-conserved pocket adjacent to the SAM/SIN/SAH binding site was also observed [34].

Various inhibitors of flavivirus MTases have been found through the use of a variety of techniques including cell-based assay, virtual screening, and structure-based design [15, 21, 22, 24–30, 39]. Although many inhibitors were found to inhibit the N7 and/or 2'-O MTase activities with *IC*_*50*_ values in the micromolar or nanomolar range (*IC*_*50*_: compound concentration required to inhibit 50% of enzyme activity), the majority of these compounds have not shown antiviral efficacy. Only a few of these compounds were found to inhibit the growth of various flaviviruses with an *EC*_*50*_ in the low micromolar range (*EC*_*50*_: effective concentration of compound to inhibit virus growth by 50%) [27, 28, 30]. However, they display relatively low potency, high cytotoxicity, and/or low therapeutic index.

To search for novel and potent MTase inhibitors, we performed virtual screening of the Diversity Set II library of 1,364 compounds from the National Cancer Institute Developmental Therapeutics Program (NCI DTP). Functional analysis indicated that two compounds, NSC 306711 and NSC 610930, inhibited both the N7 and 2’-O MTase functions. Cytotoxicity and antiviral analyses indicated that they also inhibited the virus growth with low micromolar *IC*_*50*_ in cell culture. Particularly, compound NSC306711 displayed high therapeutic index.

## Results

### Virtual screening to identify novel potent inhibitors of flavivirus MTase

**Fig 1:**
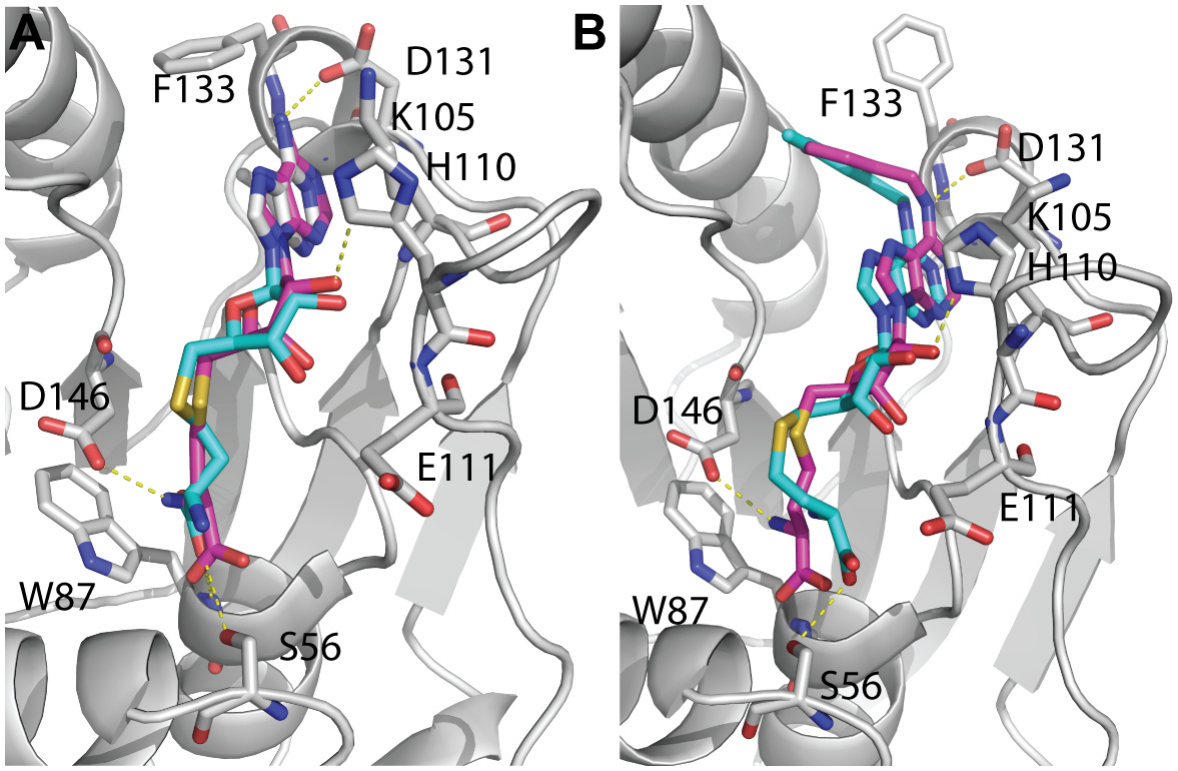
Comparison of experimentally determined and docked conformations of SAH (A) and the SAH-based inhibitor 36A (B) in the SAM-binding pocket of the DENV3 MTase. The MTase was in cartoon representation in grey color with representative contact residues in stick representation. Ligands (SAH or 36A) were in stick representation. Colors for atoms unless specified: oxygen, red; nitrogen, blue; carbon for MTase residues, grey; carbon for ligands (crystallography-determined), magenta; carbon for ligands (docked), cyan.

**Fig 2:**
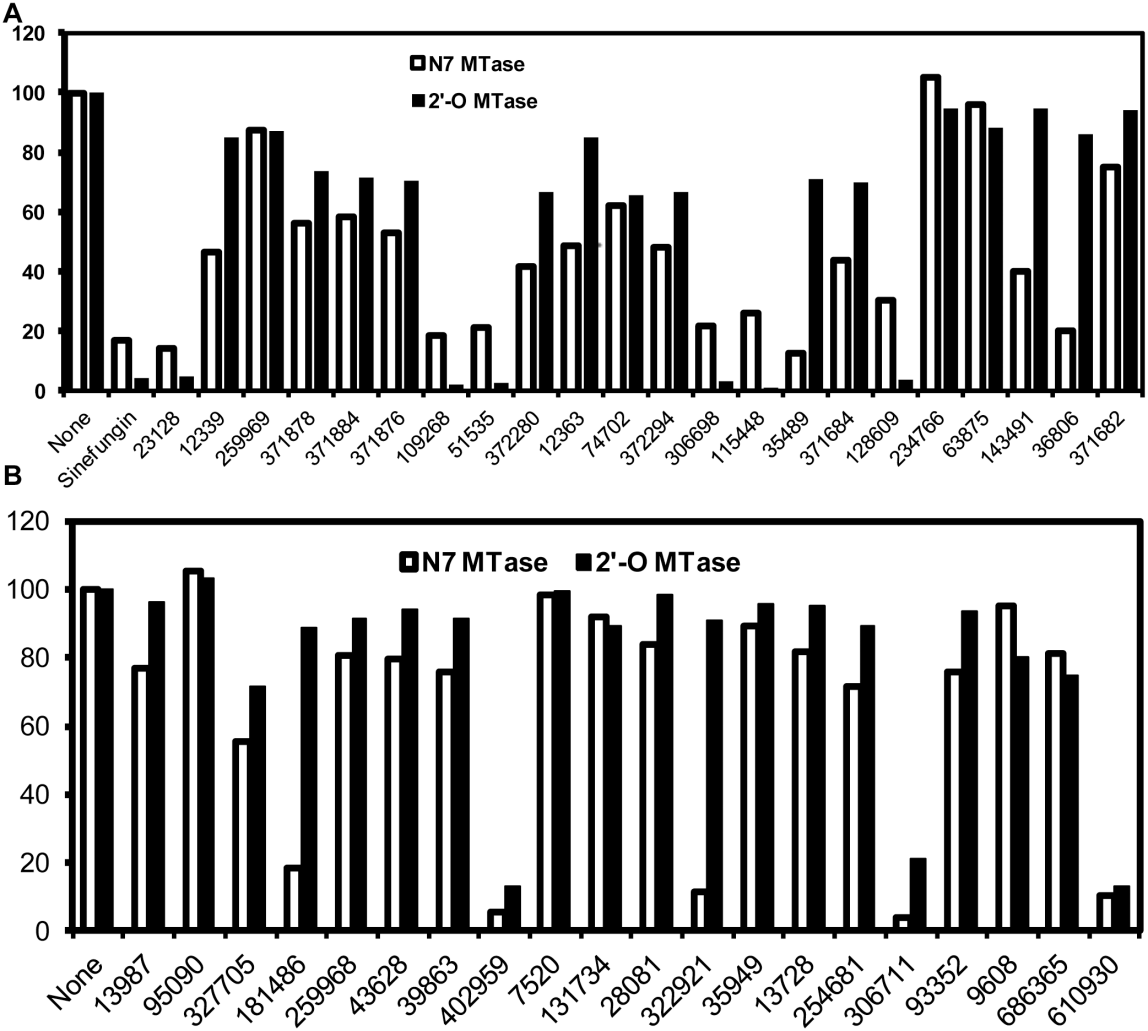
Inhibition of the N7 and 2’-O methylation activities of the WNV MTase by 42 top ranking compounds at 150 μM concentration. Inhibitions of the N7 and 2’-O methylation activities of the WNV MTase were analyzed on TLC plates. The N7 methylation was measured by conversion of G*pppA-RNA→m^7^G*pppA-RNA; the 2’-O methylation was measured by conversion of m^7^G*pppA-RNA→m^7^G*pppAm-RNA (the asterisk indicates that the following phosphate is ^32^P labeled; the RNA represents the first 90 nucleotides of the WNV genome). The spots representing different cap structures on TLC plates were quantified by a PhosphorImager. The relative methylation activity without compounds was set at 100%, and the relative methylation activity with a particular compound was defined as specific activity (compound)/specific activity (no compound) * 100.

A suitable ligand binding pocket for virtual screening (VS) is provided by the crystal structures for SAH and 36A ligands bound to the DENV3 MTase (PDB ID: 3P8Z) [39]. The DENV3 MTase-inhibitor co-structure was chosen because the SAH-derivative inhibitor occupied a flavivirus-conserved pocket [34] and clearly defined the co-factor binding pocket [39]. We first optimized the docking parameters for AutoDock Vina by re-docking SAH and 36A into the SAM-binding site of the MTase. The root-mean-square deviation (RMSD) between the re-docked and crystallography-determined conformations of SAH and 36A was 1.2 Å and 1.7 Å, respectively (fig 1). These numbers are comparable to the ones published previously, by using different structures as models [25–27]. We then applied these optimized parameters to dock the NCI diversity set II library into the binding sites of both monomers in the DENV3 MTase structure, using AutoDock Vina. We selected 42 top-ranked compounds with better scores than the SAH control for further investigation (fig 2).

### Inhibition assay

**Fig 3:**
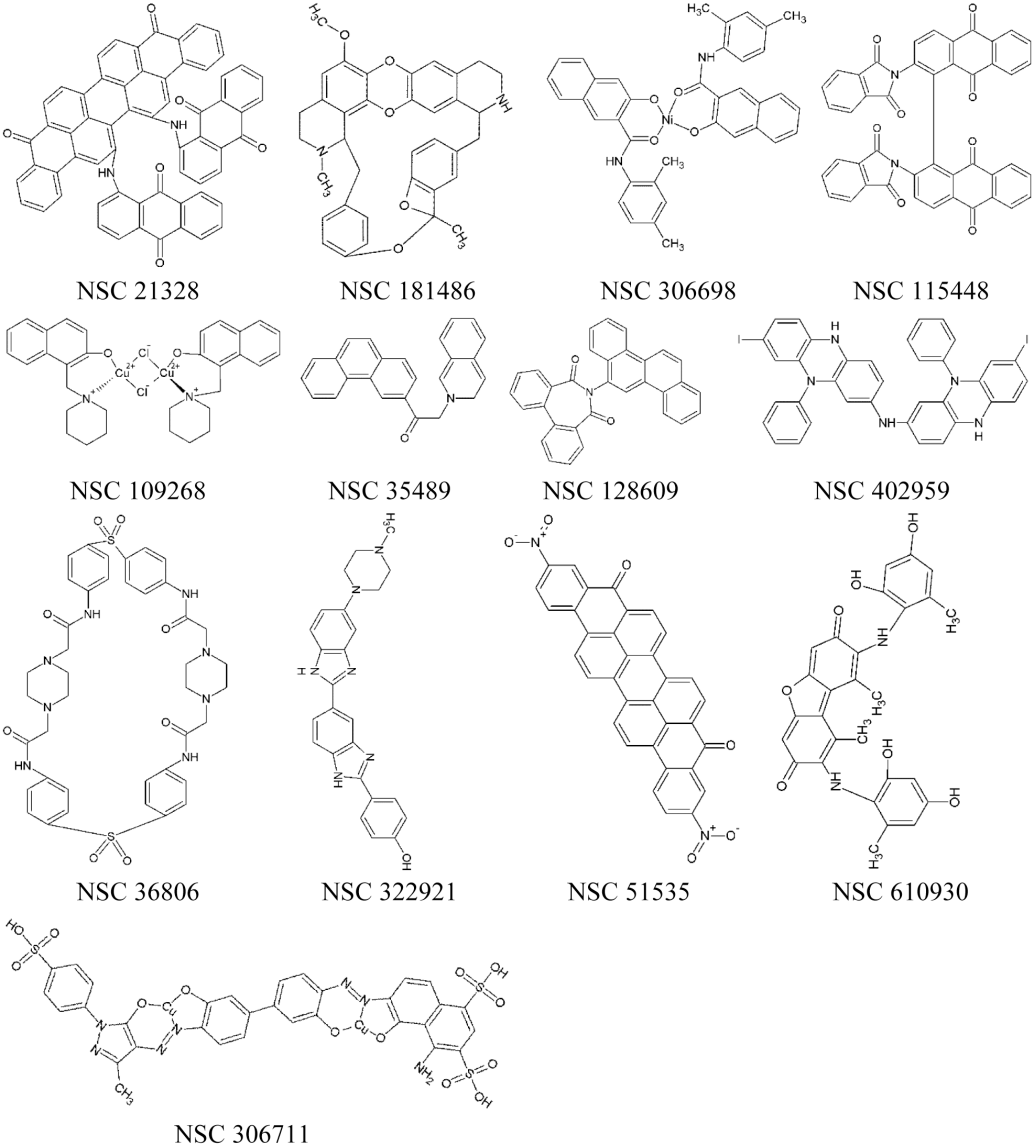
Schematic formulas of selected compounds showing *in vitro* anti-MTase activities.

Using the WNV MTase as a model, we measured both the N7 and 2’-O MTase activities of the WNV MTase in the presence of the 42 top-ranked compounds at a concentration of 150 μM with SIN as a positive control. As shown in fig 2, the positive control inhibitor SIN efficiently inhibited (~80%) the N7 activity of the WNV MTase. At 150 μM concentration, 13 out of the 42 compounds inhibited the WNV MTase N7 MTase activity by more than 60% (figs 2 and 3). Compared to the inhibition of the N7 MTase activity, the 2’-O inhibition by these compounds varied (fig 2). Similar variations of inhibitions of the N7 and 2’-O MTase activities by identical compounds have been observed previously [26, 30]. For example, SAH was reported to require 6-fold lower *IC*_*50*_ concentration for inhibitions of 2’-O than of N7 [39]. As only the N7 MTase activity is essential for the virus replication [10, 33], these 13 compounds were chosen for further analyses, although some of them showed no inhibition towards the 2’-O MTase activity.

**Fig 4:**
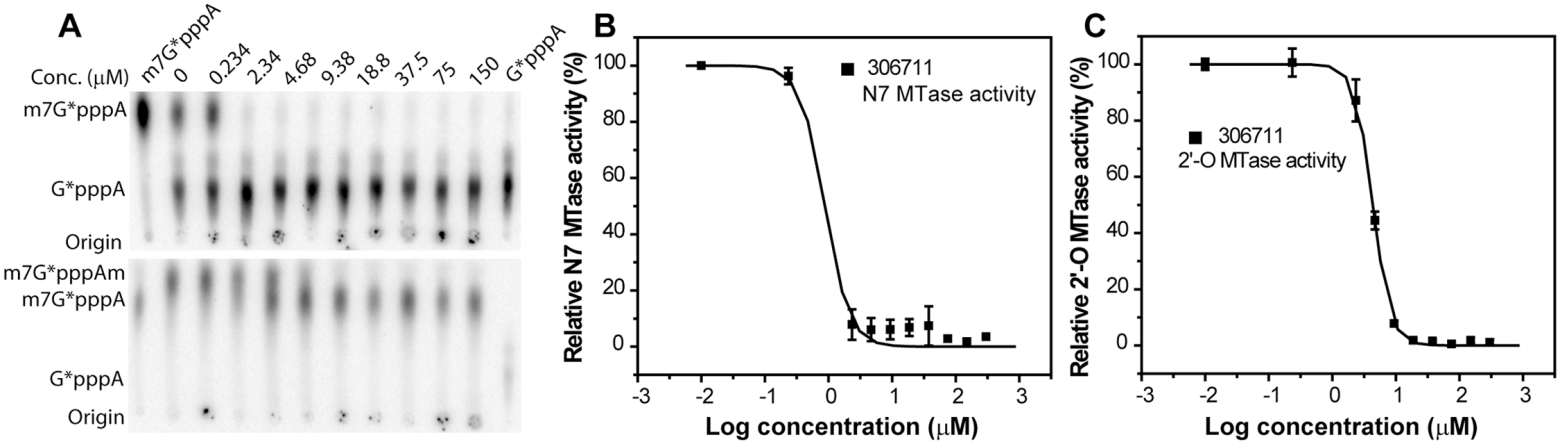
Dose response inhibition of the N7 and 2’-O methylation activities of the WNV MTase by the most potent compound NSC 306711. **(A)** TLC analyses of the N7 and 2’-O inhibition of the WNV MTase by NSC 306711. The migration positions of the G*pppA and m^7^G*pppA molecules are labeled on the side of the TLC images. The specific activity (%) for N7 = Intensity (m^7^G*pppA)/(Intensity (G*pppA)+Intensity (m^7^G*pppA)) *100). The specific activity (%) for 2’-O = Intensity (m^7^G*pppAm)/(Intensity (m^7^G*pppA)+Intensity (m^7^G*pppAm)) *100). The relative methylation activity without compounds was set at 100%, and the relative methylation activity with a particular compound was defined as specific activity (compound)/specific activity (no compound) * 100. **(B-C)** Curve fitting to determine the IC_50_ values for each compound on the N7 **(B)** and 2’-O **(C)** MTase activities of the WNV MTase. The IC_50_ value was determined by fitting of the dose–response curve as described in methods section. Each reaction was carried out in triplicate and the standard deviation is plotted.

We carried out detailed inhibition analyses of these compounds to determine their *IC*_*50*_ values for both the N7 and 2’-O activities of the WNV MTase (Table 1, Fig 4). In the absence of detergent, the anti MTase potency (*IC*_*50*_) for these compounds ranged from 0.87 μM to 95 μM for the N7 inhibition. To rule out non-specific promiscuous inhibitors [40, 41], we also carried out the N7 inhibition experiment for selected non-toxic compounds (see *CC*_*50*_ below) in the presence of detergent CHAPS (Table 1). The 2’-O inhibition was only performed in the presence of CHAPS, resulting *IC*_*50*_ from 4.3 μM to over 300 μM. Fig 4 shows the results of an example dose-response experiment of the best inhibitor, NSC 306711, for both N7 and 2’-O inhibitions (both with CHAPS). Two compounds (NSC 23128 and 115448) were excluded from further analyses as the *IC*_*50*_ values of these compounds in the presence of detergent were significantly higher than those in the absence of detergent. In addition, compound NSC35489 was also excluded due to the weak inhibition activity. All other compounds, including the most active compound NSC 306711, showed similar *IC*_*50*_ values with/without CHAPS, indicating that they are likely specific inhibitors. They were chosen for further investigations including cell-based cytotoxicity and antiviral potency analyses.

**Table 1:** Results of activity assays (*IC*_*50*_, *CC*_*50*_, *EC*_*50*_, Therapeutic Index (TI)) for selected compounds, with the WNV MTase.

Compound ID (NSC)	IC_50_ N7 (μM) No CHAPS	IC_50_ N7 (μM) with CHAPS	IC_50_ 2’-O (μM) with CHAPS	CC_50_ (μM)	EC_50_ (μM)	Therapeutic index^a^
23128	15	108	5.7	273	n.d.	
109268	16.5	n.d.^b^	6.8	11	n.d.	
51535	3.9	n.d.	5.6	5.7	n.d.	
306698	45	n.d.	49.1	32	n.d.	
115448	15.7	225	16.6	155	n.d.	
35489	95	n.d.	104	n.d.	n.d.	
128609	40	n.d.	101	192	n.d.	
36806	39	35.5	>300	362	>200	~1
181486	25.9	34.7	>300	4.8	n.d.	
402959	9.8	30.8	6.2	14.6	n.d.	
322921	29.9	26.5	>300	>500	>200	~2.5
306711	0.87	2.3	4.3	332	1.0	332
610930	4.2	18.2	5.6	117	12.6	9.3

^a^Therapeutic index was calculates as *CC*_*50*_/*EC*_*50*_;

^b^n.d., not determined.

### Cytotoxicity and antiviral analyses

**Fig 5:**
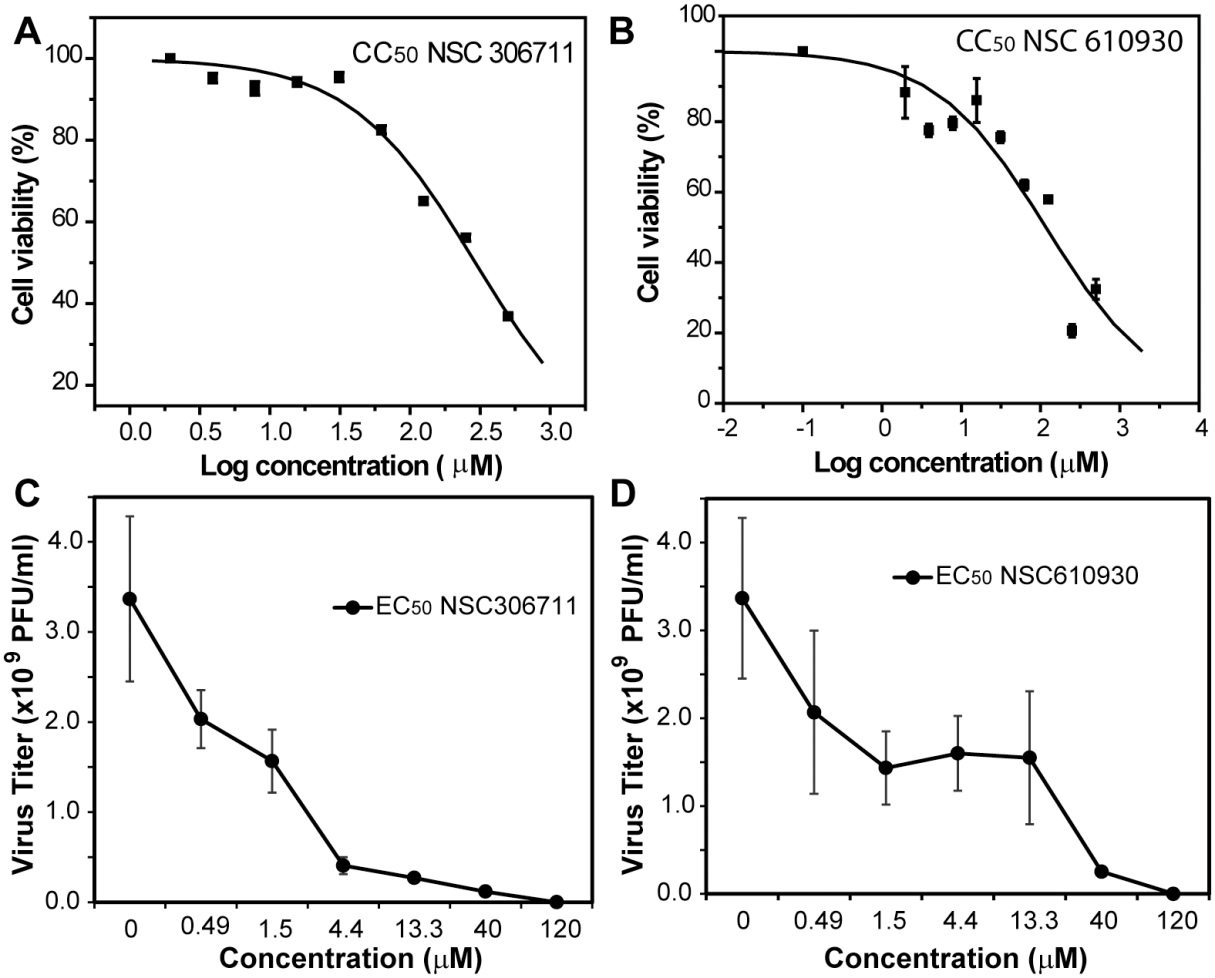
Cytotoxicity and antiviral analyses for compounds NSC 306711 and 610930. **(A & B) Cytotoxicity of NSC 306711 (A) and 610930 (B).** BHK-21 cells were incubated with various concentrations of the compound and then assayed for viability at 42 hours post-incubation. **(C & D) Inhibition of viral replication by NSC 306711 (C) and 610930 (D).** BHK cells were infected with WNV at a multiplicity of infection of 0.1, in the presence or absence of compounds. At 42 hours post-infection, viral titers in culture fluids were quantified by plaque assays on Vero cells. Each reaction was carried out in triplicate and the standard deviation is plotted.

Cell-based assays were next performed to evaluate the biological activities of the selected compounds. The cytotoxicity of these compounds was first evaluated by using a MTT cell proliferation assay with a BHK-21 cell line (Table 1, fig 5), as we described previously [20, 30]. As shown in fig 5 and Table 1, several compounds were quite toxic to the cells with the *CC*_*50*_ values similar or less than their *in vitro IC*_*50*_ values. The rest of the compounds, including NSC 36806, 322921, 306711, and 610930, showed much less toxicity, with *CC*_*50*_ values nearly 10 times higher than those of *IC*_*50*_ values. Therefore, these compounds were further investigated for their *in vitro* antiviral efficacy.

Viral titer reduction assays were used to evaluate the compounds’ antiviral efficacy. As shown in fig 5 and Table 1, compounds NSC 36806 and 322921 did not inhibit the WNV titer at the highest concentration (200 μM) tested, indicating that they are less likely to be good inhibitors for flaviviruses. In contrast, compounds NSC 306711 and 610930 clearly reduced the WNV titer in a dose-dependent manner, with *EC*_*50*_ values of 1.0 μM and 12.6 μM, respectively (fig 5). Compared to their *CC*_*50*_ values, the low *EC*_*50*_ values indicated that these two compounds display relatively good therapeutic window (Table 1). In addition, the antiviral potency of these compounds are consistent with their *IC*_*50*_ values.

### Broad spectrum anti-MTase activity

**Fig 6:**
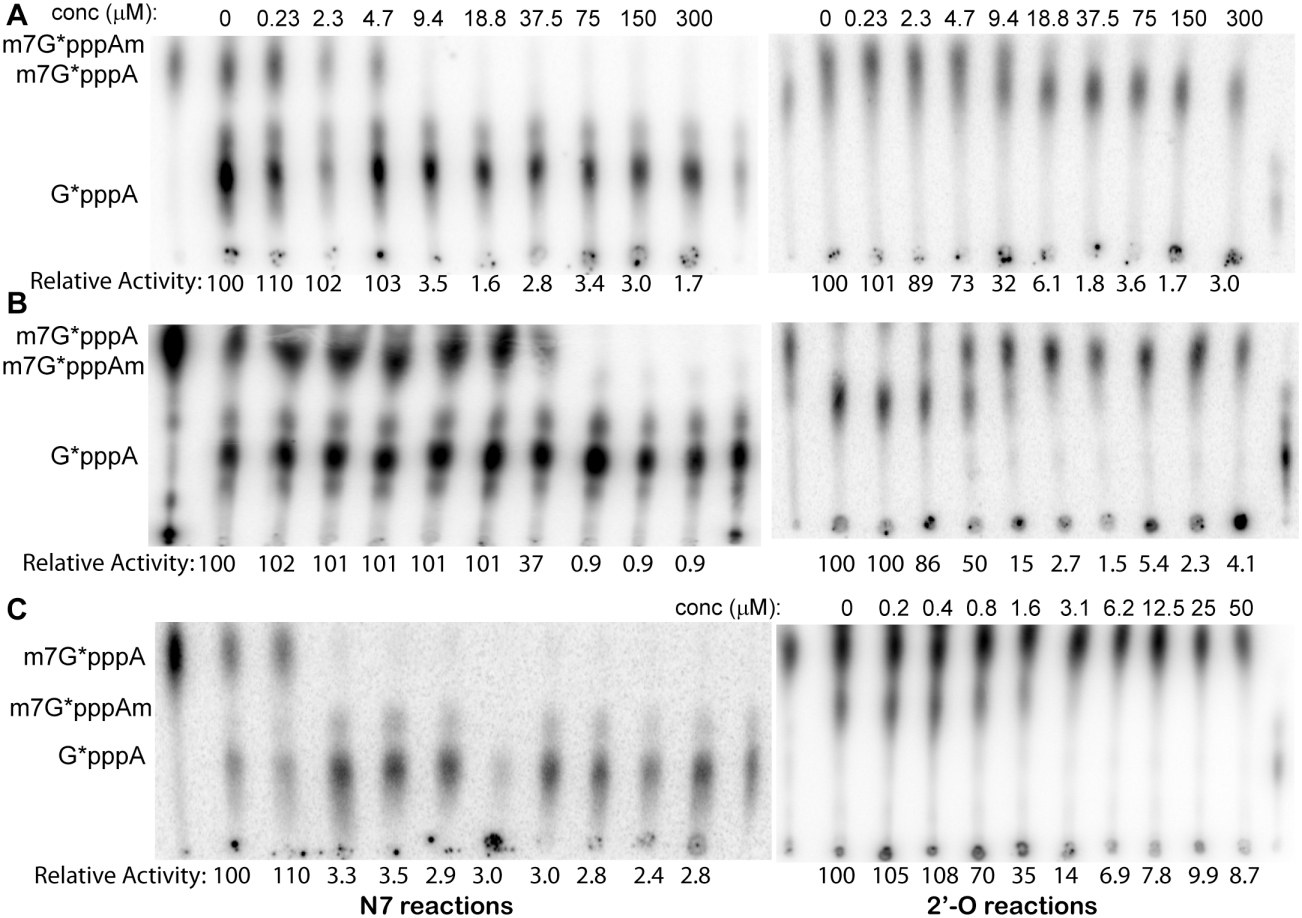
Inhibition of the MTase activities by NSC 306711. **(A-C)** TLC analyses of the dose response of NSC 306711 in inhibition of the N7 and 2’-O activities of the DENV2 **(A)**, DENV3 **(B)** and YFV **(C)** MTases. The activities without compounds were set to 100. Relative activities with each compound at a particular concentration were marked under each reaction.

Since the SAM-binding VS target site is conserved among flavivirus MTases [34], a nanomolar inhibitor targeted to this site has the potential to show broad spectrum anti-MTase activity. Therefore, we carried out inhibition assays using the recombinant MTases from DENV2, DENV3, and YFV. We noticed that the 2’-O reaction product m^7^G*pppAm migrated to different positions in these experiment (fig 6A, 6B and 6C). This was due to a known effect of nuclease P1 used in the experiment [20, 42]. Due to unknown reasons, when nuclease P1 from US Biological was used, the double methylated product would migrate to a position between G*pppA and m^7^G*pppA as shown in fig 6B and 6C, whereas it would migrate to a position above m^7^G*pppA as shown in fig 6A when nuclease P1 from SIGMA-Aldrich was used [20, 42]. Our results indicated that the compounds NSC 306711 and NSC 610930 inhibited the MTases from DENV2, DENV3, and YFV in a dose-dependent manner (fig 6, Table 2, and data not shown). Except for the DENV3 MTase, which was inhibited with a moderate *IC*_*50*_ value (36 μM) for the N7 activity, both the N7 and 2’-O activities of the DENV2 and YFV MTases were strongly inhibited by NSC 306711 with *IC*_*50*_ values in low micromolar range (Table 2). NSC 610930 also inhibited these MTases with *IC*_*50*_ values comparable to those for the WNV MTase. Overall, our results indicated that the potent compounds NSC 306711 and NSC 610930 are broad spectrum inhibitors for flavivirus MTases.

**Table 2:** Inhibition of flavivirus MTases by compounds NSC 306711 and NSC 610930.

	NSC 306711		NSC 610930	
MTases	IC_50_ N7 (μM)	IC_50_ 2’-O (μM)	IC_50_ N7 (μM)	IC_50_ 2’-O (μM)
DENV2	5.3	6.9	28	2.3
DENV3	36	4.7	32	21
YFV	1.2	1.1	20	15

### Analysis of NSC306711 and NSC610930 binding to the DENV MTase

**Fig 7:**
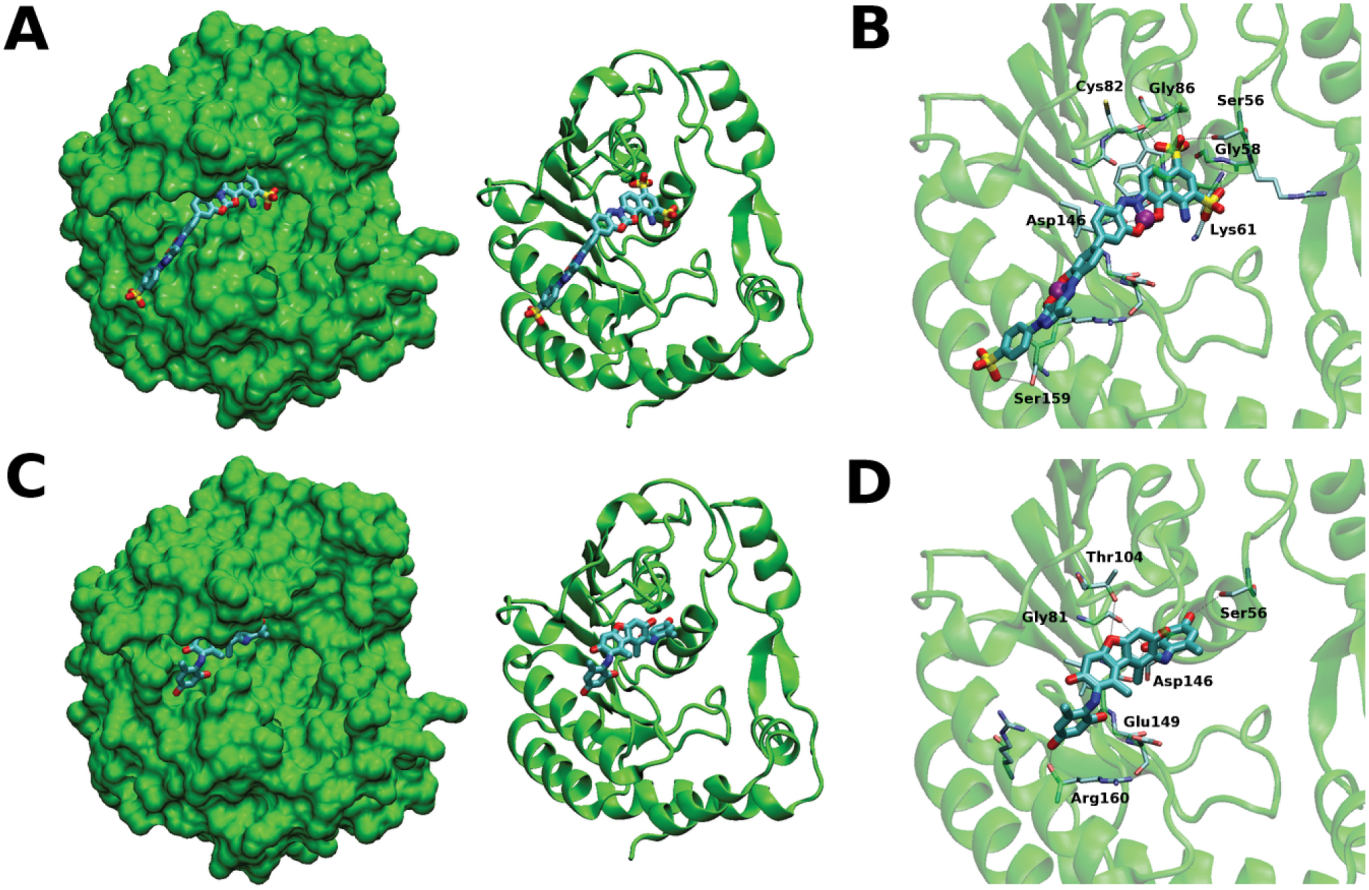
NSC 306711 and 610930 binding to the SAM-binding pocket of the DENV3 MTase. Predicted poses of compounds 306711 and 610920 in the DENV3 MTase SAM-binding pocket. **(A)** Compound 306711 orientation with respect to the full protein structure (in surface depiction on left and cartoon depiction on right); **(B)** Binding pocket interactions for compound 306711; **(C)** Compound 610920 orientation with respect to the full protein structure (in surface depiction on left and cartoon depiction on right); **(D)** Binding pocket interactions for compound 610920. Labels indicate protein amino acid residues that can possibly form H-bonds (black-dotted lines) with the ligands in these poses. Cu atoms in compound 306711 are shown as purple spheres.

The docked conformations in the SAH/36A binding site on the DENV MTase of the top two inhibitors identified (NSC306711 and NSC610930) were examined to understand their binding abilities (fig 7A and 7C). Both compounds fit into the binding pocket with multiple electrostatic and non-polar contacts with the enzyme (as indicated by atoms present within 3.5 Å). There are seven electrostatic contacts between NSC306711 and the MTase, specifically with the sidechains of residues Ser56, Lys61, and Ser159, and the backbone of residues Gly58, Cys82, Gly86, and Asp146 (fig 7B). The larger size of this compound allows it to extend out of the pocket and drape over a helical scaffold, making close contacts with 14 amino acid residues from the enzyme (shown as sticks and surfaces in fig 7B). NSC610930 has six electrostatic contacts with the MTase, with sidechains of residues Ser56 and Thr104, and backbones of residues Gly81, Asp146, Glu149, and Arg160 (fig 7D). Due to its smaller size, it is nestled in the binding pocket and makes close contact with only 9 enzyme residues (shown as sticks and surfaces in fig 7D). The larger number of electrostatic and non-polar contacts between NSC306711 and the enzyme can explain its higher inhibitory capacity as compared to NSC610930.

There are commonalities and differences between the backbone and sidechain motif binding to the DENV3 MTase for the four inhibitors: SAH, 36A, NSC306711, and NSC610930. Two of these inhibitors have electrostatic contacts with the Gly86, Trp87, Lys105, Lys130, and Asp146 backbone atoms. In addition, the backbone atoms of Gly58, Cys82, Val132, Glu149, and Arg160 form an electrostatic contact in at least one inhibitor. The common feature of all four inhibitors is an electrostatic contact with the sidechain of Ser56. Two inhibitors show electrostatic contacts with the Asp131 and Asp146 sidechains. In addition, the sidechains of residues Lys61, Thr104, His110, and Ser159 formed electrostatic contacts in at least one inhibitor. The first step in designing new inhibitors using the presently identified compounds as scaffolds could therefore use simple substitutions that can generate additional contacts with this pool of backbone and sidechain motif contacts in the DENV3 MTase SAM-binding pocket.

### Analysis of NSC306711 and NSC610930 binding to the WNV MTase

**Fig 8:**
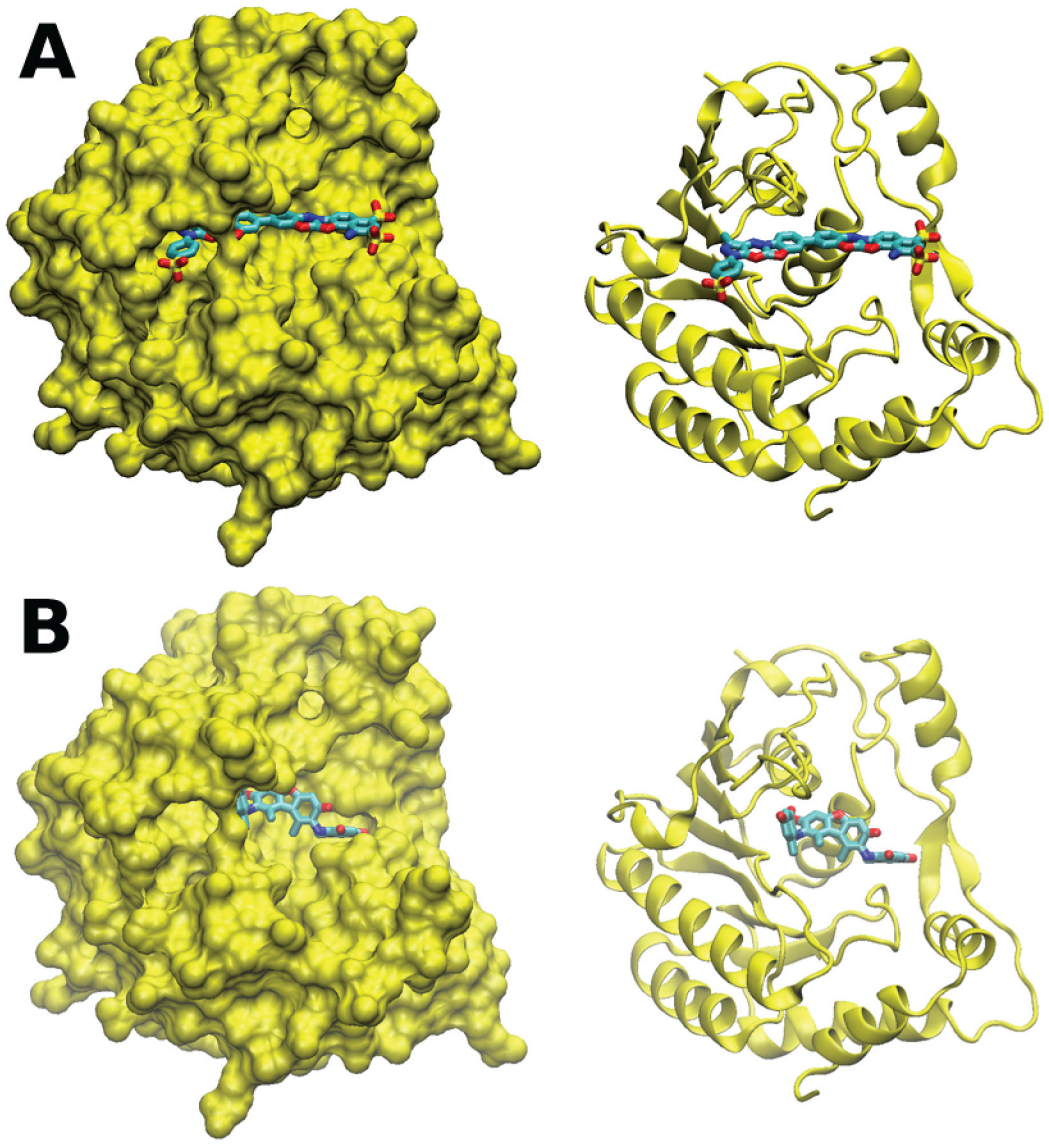
NSC 306711 and 610930 binding to the SAM-binding pocket of the WNV MTase. Predicted poses of compounds 306711 and 610920 in the WNV MTase SAM-binding pocket. **(A)** Compound 306711 orientation with respect to the full protein structure (in surface depiction on left and cartoon depiction on right); **(B)** Compound 610920 orientation with respect to the full protein structure (in surface depiction on left and cartoon depiction on right). Cu atoms in compound 306711 are shown as purple spheres.

We noticed that although the compounds were initially identified through docking into the SAM-binding pocket of the DENV3 MTase, it appears that the compounds are overall less active against the DENV3 MTase than against the others (Table 2). One explanation could be that because the substrate used in the assays was an authentic sequence of the WNV and it might not be optimal for the DENV3. This is particularly reasonable as the N7 function of flavivirus MTase requires distinct viral stem-loop structure for optimal reaction [43]. An alternative explanation is that the compounds may bind the MTases differently. To address this concern, we independently docked these two compounds into the WNV MTase (fig 8). The docking conformations were quite different from those for the Dengue MTase, suggesting that one explanation for the differences in activity could be attributed to different binding poses of the molecules in the two binding sites. Whether the compounds bind similarly or differently to these MTases will require mutational and biochemical experiments and/or co-crystal structure with bound inhibitor. However, these are outside the scope of the present study.

## Discussion

In this study we have identified potential inhibitors of flavivirus MTase using a virtual screening method, and further examined the efficacy of these compounds using *in vitro* and cell-based assays. Two of these compounds, NSC306711 and NSC610930, inhibited the MTase proteins of multiple flaviviruses, reduced WNV replication in a dose-dependent fashion, and were relatively non-toxic to BHK-21 cells. The comparatively larger size of NSC306711, and its predicted interaction with MTase residues outside of the SAM binding pocket, may be responsible for its high potency. It is possible that these “extra” interactions outside of the SAM binding pocket could be used as virtual screening parameters to identify inhibitors specific for flavivirus, but not host, MTase proteins.

A challenge to developing inhibitors specific to flavivirus MTase enzymes is the similarity between flaviviral MTases and those of the host cell. Due to the similarity of RNA, GTP, and SAM binding sites of flavivirus and host MTases, inhibitors targeted towards any of these sites may also inhibit host cell MTases and result in toxicity [44]. One difference from host MTases is the presence in flavivirus MTase proteins of an extended cleft continuing from the SAM binding pocket [34]; several inhibitory compounds that project into this cleft have been described [39]. Additionally, residues outside of the SAM binding site may confer specificity as appears to be the case with NSC306711.

A second difference is that host cells divide the N7 and 2’-O methylations among multiple enzymes, whereas flavivirus MTase proteins carry out both functions. One model of flavivirus MTase function posits a translocation of the RNA from an N7 binding position to 2-O’ binding position on the same MTase molecule during the methylation process [2, 44]. If such a translocation does occur, a small molecule or RNA analogue that blocks this process could prove a viable inhibitor. A previous study exploring compounds that bind in one of the two identified MTase RNA binding sites identified compounds with potency, but not specificity [26].

A potential third route of flavivirus MTase inhibition is to target the GTP binding site using nucleoside analogs to prevent the binding of the capped portion of the viral RNA and its subsequent methylation. Ribavirin, a nucleoside analog used clinically to treat various RNA virus infections, has been shown to bind to the DENV MTase GTP binding site and inhibit RNA cap methylation *in vitro* [22]. Interestingly, we have identified nucleoside analogs that appear to bind to both the GTP binding site as well as the SAM binding pocket, inhibiting MTase activity *in vitro* and viral replication [30]. These compounds, along with those identified in this study, give us further insight into the chemical scaffolds most likely to inhibit flavivirus MTase proteins.

## Materials and Methods

### Compounds

Compounds were obtained from the NCI DTP Open Chemical Repository (http://dtp.nci.nih.gov). [α-32P]GTP was purchased from MP Biomedicals.

### Virtual screening

The program Autodock Vina [45] was used for the molecular docking of the NCI diversity set II library obtained from the http://dtpsearch.ncifcrf.gov/FTP/DIVERSITY web address in January 2011. The sdf format library was converted to pdb format using the program babel [46]. The two DENV3 MTase monomers bound to either SAH or 36A (an SAH-derivative inhibitor, PDB ID 3P8Z) [39] were used as the target proteins. A ligand box extending 30 Å in each direction with its center located at the SAH binding site, and an exhaustiveness parameter of 8 was used for the docking. These parameters were chosen based on their ability to dock SAH or 36A (a SAH-based inhibitor) into their correct binding orientations in the target site. The predicted binding energy for SAH according to the Autodock Vina scoring function (-7.2 kcal/mol) was used as a cutoff for top-scoring compounds to test experimentally.

### Expression and purification of the NS5 MTase from WNV, YFV, DENV2 and DENV3

Recombinant MTases from WNV, YFV, DENV2 and DENV3 containing the N-terminal 300, 266, 265, and 272 amino acids of NS5 protein, respectively, were expressed and purified as described previously [20].

### *In vitro* MTase inhibition assay

The in vitro MTase inhibition assay was performed, using the 5’-end-labeled substrates G*pppA-RNA and m^7^G*pppA-RNA, representing the first 90 nucleotides of the WNV genome (the asterisk indicates that the following phosphate is ^32^P labeled), as described previously [20, 30]. The N7 and 2'-O methylation inhibition assays were performed as described previously with the addition of 0.05% CHAPS [10, 21]. To rule out none specific inhibitors, N7 inhibition experiment without CHAPS was also performed. The N7 methylation was evaluated by conversion of G*pppA-RNA→m^7^G*pppA-RNA. The 2’-O methylation was assayed by conversion of m^7^G*pppA-RNA→m^7^G*pppAm-RNA. The specific activity (%) for N7 was defined as Intensity (m^7^G*pppA)/(Intensity (G*pppA)+Intensity (m^7^G*pppA)) *100). The specific activity (%) for 2’-O was defined as Intensity (m^7^G*pppAm)/(Intensity (m^7^G*pppA)+Intensity (m^7^G*pppAm)) *100). The relative methylation activity without compounds was set at 100%, and the relative methylation activity with a particular compound was defined as specific activity (compound)/specific activity (no compound) * 100. The *IC*_*50*_ value, unless specified, was determined by fitting of the dose–response curve using the ORIGIN software package.

### Cytotoxicity assay

Cytotoxicity was measured using BHK-21 cells by a MTT cell proliferation assay using the 3-(4,5-dimethylthiazol-2-yl)-2,5-diphenyl tetrazolium bromide method (ATCC), as described previously [20, 30].

### Antiviral assay

A viral titer reduction assay was used to determine the compounds’ effect on WNV, as described previously [20, 30].
